# Indirect Impact of PD-1/PD-L1 Blockade on a Murine Model of NK Cell Exhaustion

**DOI:** 10.3389/fimmu.2020.00007

**Published:** 2020-02-11

**Authors:** Maite Alvarez, Federico Simonetta, Jeanette Baker, Alyssa R. Morrison, Arielle S. Wenokur, Antonio Pierini, Pedro Berraondo, Robert S. Negrin

**Affiliations:** ^1^Blood and Marrow Transplantation, Stanford University School of Medicine, Stanford, CA, United States; ^2^Program of Immunology and Immunotherapy, Cima Universidad de Navarra, Pamplona, Spain; ^3^Instituto de Investigación Sanitaria de Navarra (IDISNA), Pamplona, Spain; ^4^Centro de Investigación Biomédica en Red de Cáncer (CIBERONC), Madrid, Spain

**Keywords:** NK, exhaustion, chronic stimulation, PD-1/PD-L1 pathway, CD8

## Abstract

The induction of exhaustion on effector immune cells is an important limiting factor for cancer immunotherapy efficacy as these cells undergo a hierarchical loss of proliferation and cytolytic activity due to chronic stimulation. Targeting PD-1 has shown unprecedented clinical benefits for many cancers, which have been attributed to the prevention of immune suppression and exhaustion with enhanced anti-tumor responses. In this study, we sought to evaluate the role of the PD-1/PD-L1 pathway in murine natural killer (NK) cell activation, function, and exhaustion. In an *in vivo* IL-2-dependent exhaustion mouse model, neutralization of the PD-1/PD-L1 pathway improved NK cell activation after chronic stimulation when compared to control-treated mice. These cells displayed higher proliferative capabilities and enhanced granzyme B production. However, the blockade of these molecules during long-term *in vitro* IL-2 stimulation did not alter the progression of NK cell exhaustion (NCE), suggesting an indirect involvement of PD-1/PD-L1 on NCE. Given the expansion of CD8 T cells and regulatory T cells (Tregs) observed upon acute and chronic stimulation with IL-2, either of these two populations could influence NK cell homeostasis after PD-L1/PD-1 therapy. Importantly, CD8 T cell activation and functional phenotype were indeed enhanced by PD-1/PD-L1 therapy, particularly with anti-PD-1 treatment that resulted in the highest upregulation of CD25 during chronic stimulation and granted an advantage for IL-2 over NK cells. These results indicate a competition for resources between NK and CD8 T cells that arguably delays the onset of NCE rather than improving its activation during chronic stimulation. Supporting this notion, the depletion of CD8 T cells reversed the benefits of PD-1 therapy on chronically stimulated NK cells. These data suggest a bystander effect of anti-PD1 on NK cells, resulting from the global competition that exists between NK and CD8 T cells for IL-2 as a key regulator of these cells' activation. Thus, achieving an equilibrium between these immune cells might be important to accomplish long-term efficacy during anti-PD-1/IL-2 therapy.

## Introduction

Natural killer (NK) cells are a subset of innate lymphocytes that have the property of destroying target cells without prior immune sensitization in an MHC unrestricted manner (1, 2). There is strong evidence for the importance of NK cells in the eradication of cancer cells, and thus, NK cell-based therapy has been explored in a number of cancers (3–5). Indeed, the adoptive transfer of NK cells after *ex vivo* activation has proven to be safe and well-tolerated in many cancers (4). Unfortunately, clinical benefits have not been observed in all cases (2, 6). Therefore, new therapeutic strategies to fully exploit NK cell cytotoxic potential are needed.

Impaired NK cell function due to the presence of immunosuppressive cells [regulatory T cells (Tregs) or myeloid-derived suppressor cells] or cytokines (TGFβ, IL-10), downregulation of activating receptors, or increase of inhibitory receptors accounts for the limitations of NK cell-based therapy (1, 7, 8). Furthermore, NK cell exhaustion (NCE) has been identified as a self-regulatory mechanism responsible for the induction of a dysfunctional phenotype to prevent exacerbated immune responses under chronic stimulatory conditions (9). Importantly, exhaustion, described in both NK and T cells, represents a gradual process that causes a reduction in the proliferative and functional capacities of immune cells that can ultimately culminate in the elimination of the effector cells. Thus, this phenomenon has become a crucial component in the immune evasion mechanisms used by tumor and viruses to circumvent immune responses, as exhausted NK and T cells have been described after tumor exposure and chronic viral infections (7, 9–11).

An exhausted NK cell has been defined as a NK cell incapable of responding to further stimuli with downregulation of the activating transcription factors eomesodermin (Eomes) and T-box transcription factor TBX21 (T-bet), along with lower expression of activating receptors while also showing an upregulation of inhibitory receptors (7, 9, 10, 12, 13). We have recently demonstrated that the induction of the ataxia-telangiectasia mutated (ATM) DNA repair damage pathway during prolonged NK cell proliferation played a critical role in the exhaustion process (9). NKG2D downregulation, likely caused by internalization due to its binding to the stress molecule MULT1, which is upregulated upon NK activation, had a partial role in NCE as well (9). Felices et al. also showed metabolic defects in human exhausted NK cells, which were characterized by a reduction in the mitochondrial respiration profile dependent on fatty acid oxidation. This effect was prevented by mechanistic target of rapamycin (mTOR) signaling inhibition (10).

Currently, therapeutic strategies that exploit the ability of immune cells to target cancer cells have become a promising and effective approach, such as with immunomodulatory monoclonal antibodies (mAbs). Among them, mAbs that neutralize the action of checkpoint inhibitors, including PD-1 and CTLA-4 among others, have become quite popular given their tremendous success, alone or combined with other strategies, in many types of cancers (14–18). The mechanisms of action for blocking checkpoint inhibitors are mainly attributed to an increase in effector immune cells with potent antitumor responses due to a reduction of immunoregulation (14, 19).

The role of the PD-1/PD-L1 axis in the regulation of NCE, unlike in T cells (14, 20), is poorly understood, particularly in mouse NK cells (21, 22). Many studies have shown that human NK cells do, in fact, express PD-1 (23–25), but in some cases, this expression has been correlated with poor prognosis (26) and an exhausted phenotype (27, 28), whereas other studies have suggested that PD-1^+^ NK cells present a higher activation phenotype that is only suppressed by PD-L1-expressing NK cells (29). In mouse, however, the expression of PD-1 on NK cells is more restrictive, despite the fact that some studies have shown a contribution of the NK cell compartment in PD-1 blockade therapy (22, 30). According to Hsu et al., PD-1^+^ NK cells were mainly limited to the tumor microenvironment (22). Tumor-infiltrating NK cells were highly susceptible to suppression by PD-L1^+^-expressing tumor cells (22). During chronic cytomegalovirus infection in mice, we also observed an upregulation of PD-1 on NK cells, particularly in the salivary gland (9). Nevertheless, anti-PD-1 and anti-PD-L1 therapy have shown potential benefits in rescuing and/or improving NK cell functional capacities and improving anti-tumoral and anti-viral responses (26, 28, 29, 31–34), although the precise mechanisms are incompletely understood.

In this study, we aim to evaluate the role of the PD-1/PD-L1 pathway in the induction of NCE. Using an *in vivo* chronic stimulation exhaustion murine model, we found that PD-1 blockade indirectly impacts *in vivo* IL-2-induced NCE by modulating CD8 T cell expansion.

## Materials and Methods

### Mice

Wild-type C57BL/6 (H-2^b^) mice were purchased from Jackson Laboratories (Sacramento, CA) or Harlan Laboratories (Barcelona, Spain). C57BL/6 FoxP3 mutant mice expressing diphtheria toxin receptor (FoxP3 DTR) were a kind gift from Dr. Rudensky and bred in the Stanford animal facility. Female mice were used at 8–12 weeks of age and housed under specific pathogen-free conditions. All animal protocols were approved by the IACUC at Stanford University and University of Navarra.

### *In vivo* NK Cell Stimulation Model

**Figure 1:**
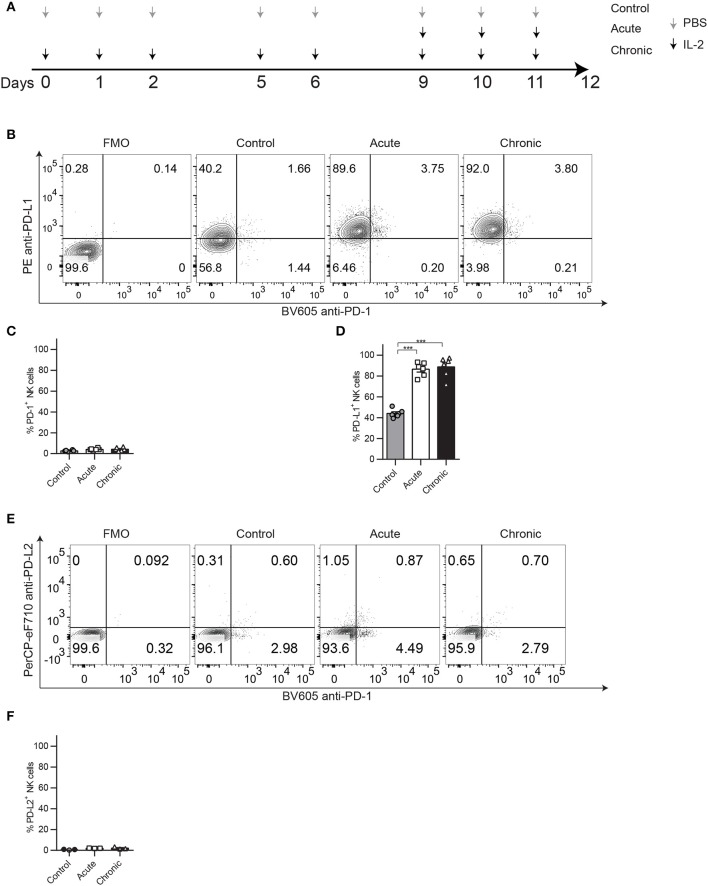
PD-1 and PD-L1 expression patterns on NK cells after chronic IL-2 stimulation. C57BL/6 were treated acutely or chronically with IL-2 or PBS (control) following the regimen dose explained in **(A)** and spleens were collected 24 h after the last treatment to analyze NK cell phenotype and function by flow cytometry. **(A)** IL-2 NCE mouse model regimen dose. **(B)** Representative dot plots on gated NK cells (CD45^+^TCRβ^−^NK1.1^+^) of PD-1 and PD-L1 are shown. **(C,D)** Total percentage of PD-1 **(C)** and PD-L1 **(D)** is shown for gated NK cells. **(E)** Representative dot plots on gated NK cells (CD45^+^TCRβ^−^NK1.1^+^) of PD-1 and PD-L2 are shown. **(F)** Total percentage of PD-L2 is shown for gated NK cells. Data are representative of five independent experiments with 3–4 mice per group (mean ± SEM). One-way ANOVA was used to assess significance. Significant differences are displayed for comparisons with the acute group ***p < 0.001).

Mice were treated with high doses (0.5–1 million IU) of recombinant human IL-2 (National Cancer Institute repository, Frederick, MD) or PBS as previously described (9) (Figure 1A). In some experiments, 200 μg of anti-PD-1 (clone RMP1-14, BioXcell, Lebanon, NH), anti-PD-L1 (clone 10F.9G2), anti-CD8β (clone Lyt 3.2, BioXcell), or control rat gamma globulin (rIgG; Jackson ImmunoResearch) was given a day prior to the beginning of IL-2 and 5 days after the first mAb treatment dose.

### *In vitro* NK Cell Stimulation Model

Single-cell suspensions from bone marrow (BM) and spleens of mice were T-cell-depleted using CD90-positive selection kit (StemCell Technology, Vancouver) according to the manufacturer's instructions and cultured in RPMI complete media at 37°C with 5% CO_2_ and with 1000 IU/ml of IL-2. On day 3 of culture, cells were treated with 20 μg/ml of anti-PD-1, anti-PD-L1 (BioXcell), or controls when indicated. Activated lymphocyte adherent killer cells, which represent 90% of purified NK cells (35), were collected at multiple time points (day 0: control, day 4: acute; days 7 and 9: chronic) to analyze NK cell activation and function. When indicated, total cells (no T cell depletion was performed) were cultured under the same conditions.

### Analysis of NK Cell Phenotype and Function

For the *in vivo* model, spleens were collected 24 h after the last IL-2 treatment and processed as previously described (9, 36). For the *in vitro* model, NK cell phenotype and function were analyzed at the indicated time points. Cell suspensions were analyzed for activating transcription factors (Eomes and T-bet), NK cell activating (NKG2D, Thy1.2, Ly49G2, DNAM-1) and inhibitory (TIGIT, CD96, PD-1, PDL-1) markers, as well as proliferation marker Ki67 as previously described (9, 36). The T cell compartment was also analyzed. Foxp3/TF staining buffer kit (eBioscience, San Diego, CA) was used according to the manufacturer's instructions. For a detailed description of the mAbs used, refer to Supplemental Table 1.

Intracellular staining was performed to detect Granzyme B (GranB) and IFNγ production after NK cell stimulation for 4 h with 10 μg/ml plate-bound anti-NK1.1 as previously described (9).

Stained cells were analyzed with an LSRII cytometer (Becton Dickinson, San Jose, CA) or Cytoflex LX (Beckmann Coulter, Indianapolis, IN). Fluorescence minus one (FMO) or biological comparison controls were used for cell analysis.

### Data Analysis

#### Principal Component Analysis

PCA was performed using the RStudio ggfortify package on the values obtained by flow cytometry. The two dominant principal components were plotted against one another to assess the relationships between the different treatments, and the PCA loading vectors (eigenvectors) were also represented.

#### T-SNE Analysis

t-Distributed Stochastic Neighbor Embedding (tSNE) dimensionality reduction algorithm was performed to analyze multiparameter flow cytometry data using Cytobank (Santa Clara, CA). Populations of T cells, MDSC, B cells, and NK cells were plotted, and the expression for Eomes and Ki67 was shown for these populations using this method.

### Statistical Analysis

Each experiment was performed at least two times with 3–4 mice per group. Student's two-tailed *t*-test, one-way ANOVA (Bonferroni post-test analysis), or two-way ANOVA (Bonferroni post-test analysis) was used when appropriate to determine statistical significance (Graphpad Prism 6, La Jolla, CA). *p*-values were considered statistically significant when *p* < 0.05.

## Results

### Impact of PD-1/PD-L1 Neutralization on NCE After Chronic IL-2 Stimulation

In a recent publication, we have demonstrated that chronic stimulation of NK cells leads to a phenotype characteristic of exhaustion defined by impaired function and downregulation of markers associated with activation and upregulation of inhibitory receptors. We showed in different models that exhaustion could be detected by the phenotypic analysis of Eomes, NKG2D, and KLRG1 expression, which was linked to reduced proliferative and functional capacities of NK cells (9). Upon IL-2-induced chronic stimulation (Figure 1A), despite not observing relevant expression of PD-1 (Figures 1B,C), an inhibitory receptor frequently associated with both NK and T cell exhaustion, we did consistently observe an upregulation of its ligand, PD-L1, compared to control-treated groups (Figures 1B,D). This phenomenon was also induced by chronic stimulation with IL-15 (Supplemental Figure 1A) or poly I:C (Supplemental Figure 1B). In contrast, PD-L2, another ligand for PD-1, was barely detected on NK cells (Figures 1E,F). Chronically stimulated NK cells also displayed reduced proliferative capacities measured by Ki67 expression (Supplemental Figure 2A) and reduced abilities to respond to NK1.1 stimulation, exemplified by lower production of granzyme b (GranB) (Supplemental Figure 2B) and IFNγ (Supplemental Figure 2C).

**Figure 2:**
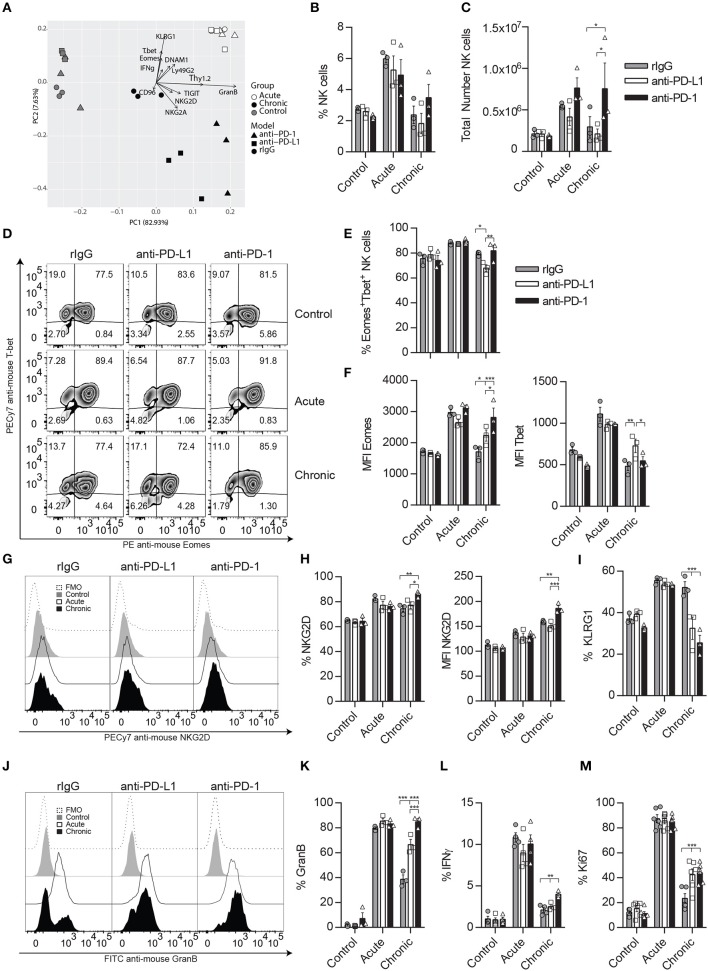
Neutralization of the PD-1/PD-L1 axis ameliorates NK cell exhaustion phenotype after chronic IL-2 stimulation. C57BL/6 mice were given two doses of anti-PD-1, anti-PD-1, or rIgG a day prior to starting IL-2 regimen dosage (Figure 1A) and 5 days after the initial mAb doses. Spleens were again collected 24 h after the last IL-2 treatment as previously explained. **(A)** Principal component analysis (PCA) of rIgG-treated (circle), anti-PD-L1-treated (square) and anti-PD-1-treated (triangle) NK cells after control (gray), acute (white), or chronic (black) IL-2 stimulation is shown. The data represent the weight that the flow cytometer analyzed NK cell parameters (loading vectors: eigenvectors) have on the PCA distribution. **(B,C)** Total percentage **(B)** and the total number **(C)** of NK cells (CD45^+^TCRβ^−^NK1.1^+^) is shown after IL-2 stimulation in the spleen. **(D–F)** Representative dot plots **(D)** and MFI **(E,F)** expression for Eomes and T-bet are shown on gated Eomes^+^ or T-bet^+^ NK cells, respectively. **(G–I)** Representative dot plots **(G)**, total percentage **(H)**, and MFI **(I)** are shown for NKG2D on gated NK cells. **(I)** Total percentage of KLRG1^+^ NK cells is shown on gated NK cells **(J–L)** Representative histograms **(J)** and the total percentage of GranB **(K)** as well as IFNγ **(L)** are shown for gated NK cells (CD3^−^CD49b^+^) after NK1.1 stimulation. **(M)** Ki67 expression is shown for gated NK cells. Data are representative of three independent experiments with 3–4 mice per group (mean ± SEM). Two-way ANOVA was used to assess significance. Significant differences are displayed for comparisons with the rIgG-treated group (*p < 0.05, **p < 0.01, ***p < 0.001).

Another important characteristic during NCE, that we have previously observed, was a drastic reduction in the total numbers of NK cells recovered after chronic stimulation when compared to acute stimulation (9). Given the immunoregulatory role of PD-1, as well as the high levels of PD-L1 surface expression on NK cells, we next evaluated the impact of the neutralization of the PD-1/PD-L1 pathway in the induction of NCE during chronic stimulation. Mice were treated with neutralizing mAbs against PD-1 or PD-L1 before IL-2 treatment, and the phenotypic and functional analysis of NK cells was determined by flow cytometry (Figure 2). A principal component analysis (PCA) of all the parameters studied revealed a clear differentiation between control and acutely stimulated NK cells driven by the expression of activating and inhibitory markers according to the PC1 and PC2 such as Eomes, T-bet, KLRG1, Thy1.2, and GranB among others with no major differences between rIgG-, anti-PD-L1-, and anti-PD-1-treated NK cells (Figure 2A, Supplemental Figures 3A,B). rIgG-treated chronically stimulated NK cells clustered closer to unstimulated NK cells as previously described (9) (Figure 2A). However, anti-PD-L1- or anti-PD-1-treated chronically stimulated NK cells were highly influenced by the parameters involved in PC1 (Thy1.2 and GranB), and their location was somewhat intermediate between chronic and acute stimulated NK cells (Figure 2A, Supplemental Figures 3A,B). These differences in the phenotype of NK cells could explain the slight increase in the percentage and the significant increase in the total number of NK cells obtained from spleens of IL-2 chronically stimulated mice after PD-1/PD-L1 blockade (Figures 2B,C), which hints toward a reduction in exhaustion.

A detailed examination of the transcription factors influencing NCE (Eomes and T-bet) showed that the percentage and median fluorescence intensity (MFI) of Eomes were significantly increased in anti-PD-L1- and/or anti-PD-1-treated NK cells (Figures 2D–F). Similarly, NKG2D percentage and MFI were also enhanced after anti-PD-1 treatment in chronically stimulated NK cells when compared to rIgG-treated NK cells (Figures 2G,H), while the percentage of KLRG1 was diminished in both anti-PD-L1- and anti-PD-1-treated chronically stimulated NK cells when compared to rIgG (Figure 2I). The expression of the inhibitory receptor Ly49G2, previously associated with an activation phenotype (37) was also significantly upregulated after chronic stimulation of anti-PD-1-treated NK cells compared to rIgG-treated NK cells (Supplemental Figures 3C,D). In agreement with a superior activation phenotype, the activation markers Thy1.2 and DNAM1 were significantly upregulated as well, particularly in the case of anti-PD-1 treatment (Supplemental Figures 3C–F). In contrast, the expression of NK cell inhibitory receptors (NKG2A, TIGIT, and CD96) was not altered by PD-1/PD-L1 inhibition (Supplemental Figures 3G–I).

Notably, *ex vivo* NK cell re-stimulation with anti-NK1.1 induced an increase of GranB production by chronically stimulated NK cells treated with anti-PD-L1 or anti-PD-1 compared to rIgG (Figures 2J,K). Indeed, GranB production in the case of anti-PD-1-treated NK cells reached levels similar to those observed during acute stimulation (Figures 2J,K), results that correlated with the lytic capacities of anti-PD-1 treated NK cells (Supplemental Figure 3J). IFNγ production was just mildly increased in anti-PD-1-treated chronically stimulated NK cells (Figure 2L, Supplemental Figure 3K). Interestingly, the phenotypic and functional changes in chronically stimulated NK cells treated with anti-PD-L1/PD-1 suggested a reduction of exhaustion, also signified by a retention in their proliferative capacities measured by an increase of Ki67 expression when compared to chronically stimulated rIgG-treated NK cells (Figure 2M).

**Figure 3:**
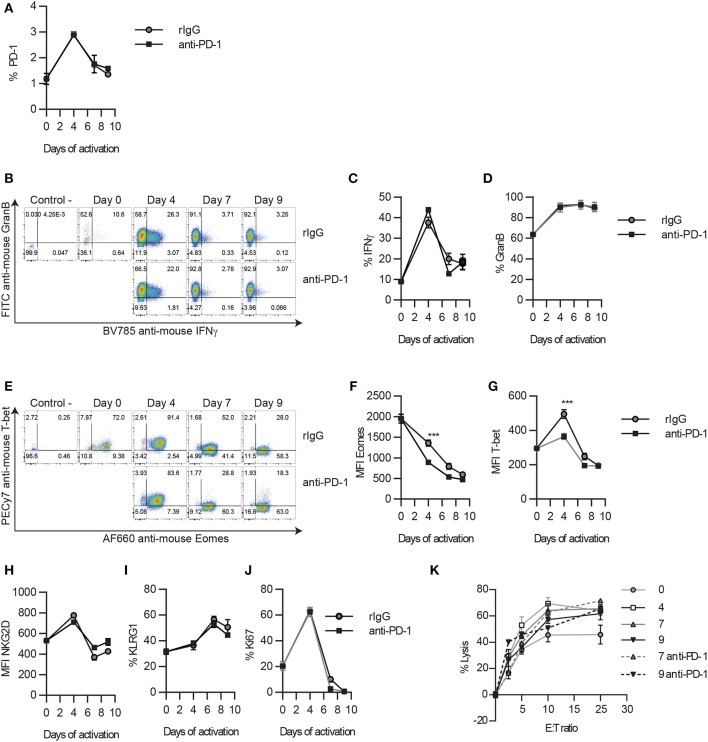
PD-1 blockade does not alter the timeline for NK cell exhaustion during *in vitro* long-term stimulation. Thy1.2^−^ NK cells were cultured with IL-2 in the presence of anti-PD-1 or isotype control, as explained in the Materials and Methods section. Adherent NK cells were collected at different time points and analyzed by flow cytometry. **(A)** The expression of PD-1 on NK cells is shown. **(B–D)** Representative dot plots **(B)**, and the total percentage of GranB **(C)** and IFNγ **(D)** producing NK cells after NK1.1 stimulation are shown. **(E)** Representative dot plots of Eomes and T-bet are shown for gated NK cells. **(F,G)** The MFI of Eomes **(F)** and T-bet **(G)** on Eomes^+^ or T-bet^+^ NK cells is shown, respectively. **(H–J)** The total percentage of the other hallmarks of NCE (NKG2D, KLRG1, and Ki67) are shown on gated NK cells. **(K)** The percentage of lysis of CFSE-labeled Yac1 cells is shown at different effector:target (E:T) ratios. Data are representative of three independent experiments done in triplicate (mean ± SEM). Two-way ANOVA was used to assess significance. Significant differences are displayed for comparisons with the rIgG-treated group (***p < 0.001).

To determine if the implication of PD-1 therapy on NCE was exerted directly, we next evaluated the impact of PD-1 neutralization during long-term *in vitro* IL-2 stimulation. Correlated with the lower expression of PD-1 detected by a non-competitive binding antibody (Figure 3A), no significant differences were observed in GranB and IFNγ production upon NK1.1 stimulation (Figures 3B–D). Accordingly, except for a small decrease on the MFI of T-bet and Eomes at the peak of activation on day 4 (Figures 3E–G), no differences on T-bet, Eomes, NKG2D, KLRG1, or Ki67 were detected after anti-PD-1 treatment during long-term *in vitro* IL-2 NK cell activation (Figures 3E–J), unlike what has previously been demonstrated with the inhibition of the ATM DNA repair damage pathway (9). NK cell lytic capacities were not altered by anti-PD-1 treatment as well (Figure 3K). Similarly, blockade of PD-L1 did not cause any changes on NK cell phenotype and function (Supplemental Figure 4). These results suggest that PD-1 does not directly affect NCE despite observing a mild reduction of exhaustion when administered *in vivo*.

### Depletion of Treg Boost CD8 T Cell Expansion and Activation

**Figure 4:**
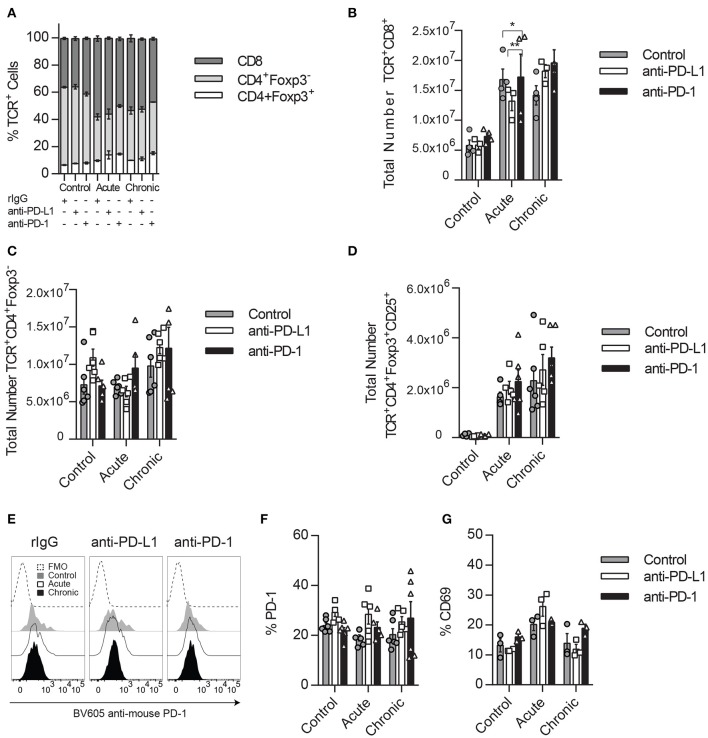
Impact of PD1/PD-L1 neutralization in the Tregs compartment. **(A)** Splenic percentage distribution of CD8 T cells (CD45^+^TCRβ^+^CD8α^+^), CD4 T cells (CD45^+^TCRβ^+^CD4^+^Foxp3^−^), and Tregs (CD45^+^TCRβ^+^CD4^+^Foxp3^+^) is shown for TCRβ^+^ cells (CD45^+^TCRβ^+^). **(B–D)** Total number of CD8 T cells **(B)**, conventional CD4 T cells **(C)**, and Tregs **(D)** collected from the spleen after IL-2 stimulation. **(E,F)** Representative histograms **(E)** and total percentage **(F)** of PD-1 expression on gated Tregs. **(G)** Total percentage of CD69 is shown on gated Tregs. Data represent one or two experiments of a total of three independent experiments with 3–4 mice per group (mean ± SEM). Two-way ANOVA was used to assess significance. Significant differences are displayed for comparisons with the rIgG-treated group (*p < 0.05, **p < 0.01).

In order to determine if a crosstalk between immune cell populations exists during PD-L1/PD-1 therapy that might influence NK cells, the T cell compartment was also evaluated. As expected, the percentage of CD8 and Tregs (TCR^+^CD4^+^Foxp3^+^) was increased during acute and chronic IL-2 stimulation, but no significant differences were detected between treated groups within each stimulatory condition (Figure 4A). When the total number of T cells was analyzed, an increase of CD8 T cells was observed during acute IL-2 stimulation, and these numbers were maintained after chronic stimulation (Figure 4B), unlike conventional CD4 T cells, whose numbers did not suffer big changes during IL-2 stimulation (Figure 4C). Similar to CD8 T cells, and equally expected, Tregs underwent a strong expansion upon IL-2 stimulation when compared to unstimulated control mice (Figure 4D). However, anti-PD-1/PD-L1 therapy did not seem to majorly alter the number of either CD8 T cells or Tregs during IL-2 treatment. When different Treg activation markers were analyzed, we observed that PD-1/PD-L1 and/or IL-2 therapy did not affect the PD-1 and CD69 expression on Tregs (Figures 4E–G).

Many studies have demonstrated that in addition to preventing CD8 T cell exhaustion, anti-PD-1/PD-L1 therapy works through the suppression of the PD-1 dependent inhibitory properties of Tregs, resulting in an increase of CD8 T cell activation and function (11, 19, 38–40). In NK cells, Tregs have also shown to suppress NK cells through a variety of mechanisms (36, 41, 42).

**Figure 5:**
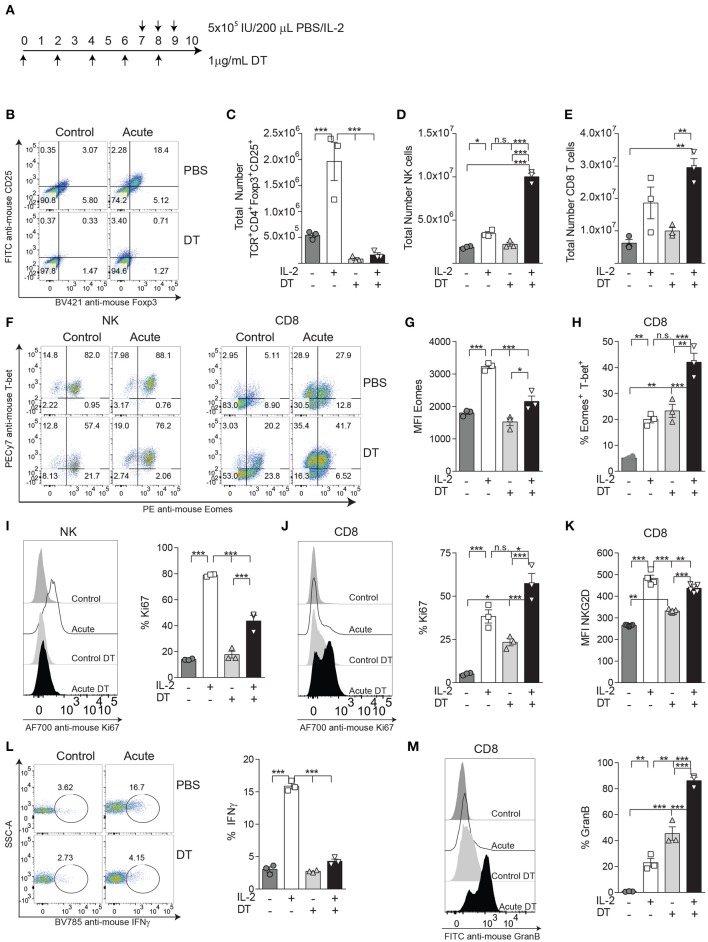
The activation status on CD8 T cells, but not on NK cells, is enhanced during IL-2 stimulation by the absence of Tregs. **(A)** The experimental regimen used to deplete Tregs on DTR-Foxp3 transgenic mice with diphtheria toxic (DT) treatment on acutely IL-2 stimulated mice. **(B)** Representative dot plots of CD25 and Foxp3 on TCRβ^+^CD4^+^ T cells after DT treatment after IL-2 stimulation. **(C–E)** Total number of Tregs (TCRβ^+^CD4^+^Foxp3^+^CD25^+^), NK cells (TCRβ^−^NK1.1^+^), and CD8 T cells (TCRβ^+^CD4^−^CD8^+^) obtained from the spleen of treated mice. **(F)** Distribution of Eomes and T-bet is shown for gated NK (left panel) and CD8 T cells (right panel). **(G)** MFI expression of Eomes on gated Eomes^+^ NK cells after acute IL-2 stimulation. **(H)** The percentage of the activating population Eomes^+^T-bet^+^ on CD8 T cells is shown. **(I,J)** The total percentage of Ki67 for NK cells **(I)** and CD8 T cells **(J)** is shown. **(K)** The MFI of NKG2D gated on NKG2D^+^ CD8 T cells is shown. **(L)** The ability of IL-2-treated NK cells to respond to NK1.1 stimulation, assessed by IFNγ production, is shown. **(M)** GranB production by IL-2-treated CD8 T cells is shown. Data are representative of two independent experiments with three mice per group (mean ± SEM). Two-way ANOVA was used to assess significance (*p < 0.05, **p < 0.01, ***p < 0.001).

In our IL-2 model, Treg expansion is likely benefited by the constitutive expression of the high-affinity IL-2Rα CD25, thus exerting stronger suppression toward CD8 T cells and NK cells. In order to explore how Tregs could affect NK and CD8 T cell distribution and activation status and see if these results correlate with the ones obtained during PD-L1/PD-1 therapy where Tregs immunosuppression efficacy might be diminished, we depleted Tregs prior to acute IL-2 stimulation using Foxp-3-expressing diphtheria toxin (DT) receptor transgenic mice, a mouse model that allows *in vivo* Treg depletion (Figures 5A–C). Under these conditions, the total numbers of both NK and CD8 T cells were dramatically augmented when IL-2 was given in the absence of Tregs (Figures 5D,E). Surprisingly, the MFI of Eomes was significantly reduced on NK cells (Figures 5F,G), whereas the proportion of CD8 T cells double-positive for Eomes and T-bet was highly enhanced (Figures 5F,H). According to an exhaustion phenotype, the percentage of NKG2D was decreased while the expression of KLRG1 was maintained at high levels on IL-2-treated NK cells stimulated in the absence of Tregs (Supplemental Figure 5). A reduction of Ki67 in NK cells (Figure 5I) but not in CD8 T cells (Figure 5J), along with an increased number of CD8 T cells expressing NKG2D MFI (Figure 5K), also indicated a higher activation status of CD8 T cells when Tregs were not present. More importantly, the lack of response to stimuli by NK cells shown by a lower production of IFNγ (Figure 5L) contrasted with the strong increase of GranB production by CD8 T cells during acute treatment in the absence of Tregs (Figure 5M). These data suggest that situations of elevated CD8 T cell activation result in a negative feedback on NK cells due to a reduction of the NK cell activation threshold, thus delaying the onset for exhaustion.

### The Inhibition of the PD-1/PD-L1 Pathway Augments CD8 T Cell Activation

**Figure 6:**
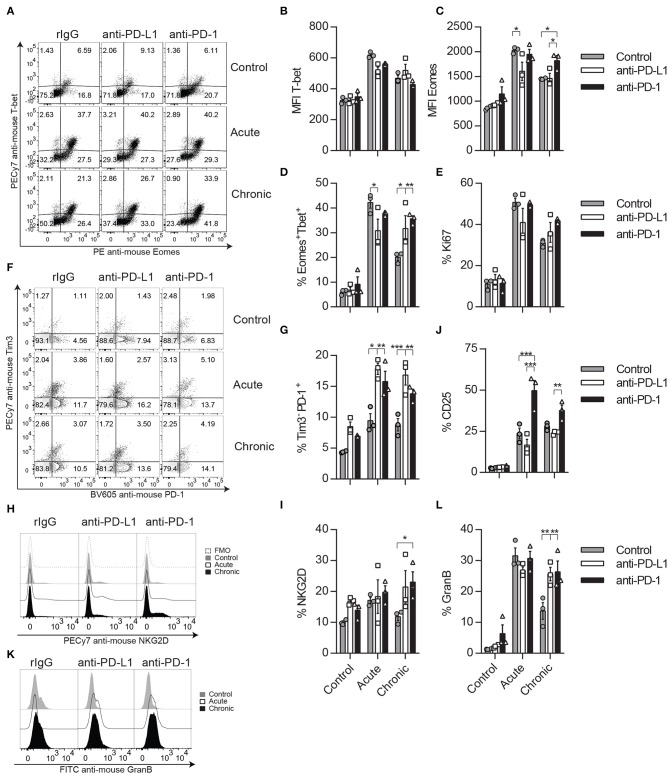
CD8 T cell activation phenotype is improved after anti-PD-L1 or anti-PD-1 treatment during chronic IL-2 stimulation. **(A–C)** Representative dot plots **(A)** and MFI **(B,C)** expression for Eomes and T-bet are shown on gated CD8 T cells (CD45^+^TCRβ^+^CD4^−^CD8α^+^). **(D)** Total percentage of Eomes and T-bet-positive CD8 T cells is shown. **(E)** Proliferative potential of CD8 T cells assessed by Ki67 expression is shown. **(F)** Representative dot plots of Tim-3 and PD-1 are shown on gated CD8 T cells. **(G)** Total percentage of Tim-3^−^PD1^+^ cells is shown on gated CD8 T cells. **(H,I)** Representative histograms **(H)** and total percentage **(I)** of NKG2D are shown for gated CD8 T cells. **(J)** Total percentage of CD25 is shown on gated CD8 T cells. **(K,L)** Representative histograms **(K)** and total **(L)** GranB production of CD8 T cells are shown. Data are representative of three independent experiments with 3–4 mice per group (mean ± SEM). Two-way ANOVA was used to assess significance. Significant differences are displayed for comparisons with the rIgG-treated group (*p < 0.05, **p < 0.01, ***p < 0.001).

Because in the absence of Tregs we have seen an improvement in the activation and functional properties of CD8 T cells, likely due to the competition for cytokines between NK and CD8 T cells, we argue that PD-1/PD-L1 blockade might result in a similar phenomenon during chronic IL-2 treatment. Therefore, the CD8 T cell activation and functional status were exhaustively evaluated after IL-2 administration in anti-PD-1- or anti-PD-L1-treated mice. Interestingly, chronic stimulation prompted a significant increase of the MFI of Eomes in CD8 T cells, as well as an increase of the total percentage of CD8 T cells expressing both Eomes and T-bet, which suggests a superior activation level at this time point in anti-PD-1-treated cells (Figures 6A–D). Despite no statistically significant differences observed in the proportion of Ki67-positive expressing CD8 T cells after PD-L1/PD-1 therapy during chronic stimulation when compared to control-treated mice, a less severe reduction of this proliferative marker between acute and chronic stimulation was observed when anti-PD-1 was administered (Figure 6E). The improvement on Eomes and T-bet expression was not associated with an increase of the central memory (CD44^+^CD62L^+^) or effector memory (CD44^+^CD62^−^) CD8 T cell populations as no significant differences were found between rIgG- and anti-PD-1-treated chronically stimulated CD8 T cells (Supplemental Figure 6A). In agreement with the results suggesting a higher activation status, an increase of a CD8 T cell population that expresses PD1 but not Tim-3 was also observed after PD-1 or PD-L1 inhibition during acute and chronic stimulation (Figures 6F,G). Furthermore, and also correlated with a higher activation status, chronically stimulated CD8 T cells treated with anti-PD-L1 or anti-PD-1 expressed significantly higher levels of the activating receptor NKG2D when compared to rIgG (Figures 6H,I). It has been previously shown that IL-2 can expand non-antigen-specific bystander memory CD8 T cells within the CD44^+^ CD8 T cell compartment that express NKG2D but lack expression of CD25, the high-affinity receptor for IL-2 (IL2R) (36, 43). However, during chronic stimulation, a decrease in this population was still observed independently of PD-1 treatment (Supplemental Figure 6B). Interestingly, when CD25 expression was evaluated on CD8 T cells, the highest increase of CD25 after IL-2 administration was observed in anti-PD-1-treated mice (Figure 6J). This phenomenon could provide a homeostatic advantage to anti-PD-1-treated CD8 T cells over NK cells in the presence of IL-2.

Notably, PD-1/PD-L1 pathway neutralization did have a functional benefit on CD8 T cells, as they expressed higher levels of GranB when treated with anti-PD-L1 or anti-PD-1 (Figures 6K,L). These results suggest that anti-PD-1 treatment does not seem to favor a specific CD8 T cell subset but rather improves overall activation and functional parameters of CD8 T cells.

### The Onset of NCE Is Restored in the Absence of CD8 T Cells

**Figure 7:**
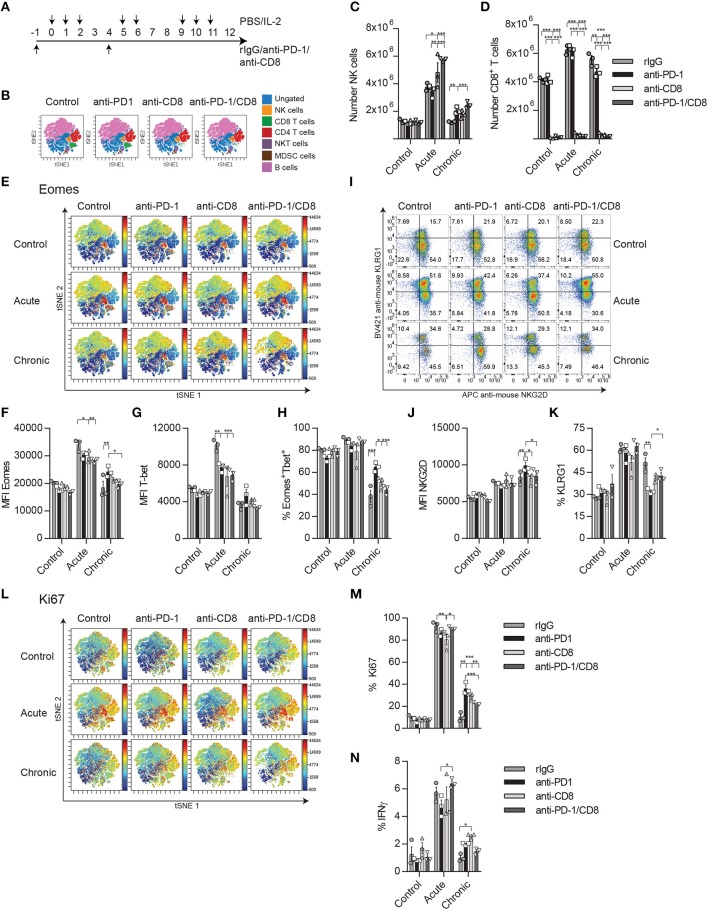
CD8 T cell depletion reverses the bystander effect of PD-1 blockade on NK cell activation during chronic IL-2 stimulation. **(A)** Regimen dose schedule followed for CD8 T cell depletion experiments. **(B)** t-SNE analysis is shown displaying the distribution of immune cell populations (ungated: blue; CD19^−^Ly6G^−^CD3^−^CD4^−^CD8^−^NK1.1^+^ NK cells: yellow; CD19^−^NK1.1^−^CD3^+^CD4^−^CD8 T cells: green; CD19^−^NK1.1^−^CD3^+^CD8^−^ CD4 T cells: red; CD19^−^CD3^+^CD8^−^NK1.1^+^CD4^+^ NKT cells: purple; CD19^−^CD3^−^Ly6G^+^CD11b^+^MDSC: brown; and CD3^−^NK1.1^−^Ly6G^−^CD19^+^ B cells: pink) after CD8 T cell depletion in unstimulated mice. **(C,D)** The total number of NK cells **(C)** and CD8 T cells **(D)** collected from the spleen after IL-2 stimulation is shown. **(E)** t-SNE analysis shows the Eomes expression of the different immune populations after IL-2 stimulation. **(F,G)** MFI expression of Eomes and the total percentage of the activating Eomes^+^T-bet^+^ NK cell population is shown on gated Eomes^+^ or T-bet^+^ NK cells (CD19^−^Ly6G^−^CD3^−^CD4^−^CD8^−^NK1.1^+^). **(H,I)** NKG2D percentage **(H)** and MFI **(I)** are shown. **(J)** The total percentage of KLRG1 on gated NK cells is shown. **(K)** t-SNE analysis shows the Ki67 expression of the different immune populations after IL-2 stimulation. **(L)** The percentage of Ki67 is shown for gated NK cells. **(M)** The percentage of IFNγ is shown on gated NK cells after NK1.1 stimulation. Data are representative of two independent experiments with three mice per group (mean ± SEM). Two-way ANOVA was used to assess significance. Significant differences are displayed for comparisons with the PD-1-treated group (*p < 0.05, **p < 0.01, ***p < 0.001).

Next, CD8 depletion studies were done in order to discern the impact of PD-1 between this immune cell population and NK cells as well as to determine if, in the absence of CD8 T cells, the IL-2-dependent induction of NCE follows the patterns previously observed (Figure 7A). This study was performed because of the negative feedback observed between NK and CD8 T cells during IL-2 stimulation in a Treg-deficient mouse model (Figure 5) and the higher activation status of CD8 T cells after PD-1/PD-L1 therapy. Therefore, a similar interaction might be driving the effect of PD-L1/PD-1 therapy on chronically stimulated NK cells, altering NK cell homeostasis and the course of NCE.

A t-SNE analysis of the flow cytometer data was used to better visualize the different immune populations affected by PD-1 blockade and CD8 T cell depletion and the changes in expression of Eomes and Ki67. After CD8 T cell depletion (Figure 7B, Supplemental Figure 7), the percentage and number of NK cells were significantly increased during both acute and chronic stimulation (Figures 7B–D). When the exhaustion parameters were studied on NK cells after chronic stimulation, a reduction of the MFI for Eomes as well as in the proportion of Eomes^+^T-bet^+^ NK cells was noted in anti-PD-1/CD8-treated mice compared to anti-PD-1 treatment (Figures 7E–H). Curiously, there were no differences between rIgG and anti-PD-1/CD8-treated NK cells in these parameters (Figures 7E–H). Similar trends were obtained for NKG2D and KLRG1, with the expected reduction of NKG2D MFI and increase of KLRG1 on NK cells, phenomena typical of exhaustion, when comparing anti-PD-1/CD8- and anti-PD-1-treated mice (Figures 7I–K). Additionally, the delay observed after anti-PD-1 treatment in the reduction of the proliferative capacities of NK cells during chronic stimulation when compared to rIgG was lost in the absence of CD8 T cells (Figures 7L,M). Furthermore, IFNγ production was mildly, although not significantly, reduced upon NK1.1 stimulation with levels of anti-PD-1/CD8-treated NK cells similar to those of rIgG-treated NK cells (Figure 7N).

The *in vitro* culture of NK cells in the presence of the T cell compartment also caused a delay on NK cell activation after long-term IL-2 stimulation with lower IFNγ and granzyme B production after NK1.1 stimulation, and lower Eomes, T-bet, and NKG2D expression at day 4 (Supplemental Figures 8A–G). Although no major differences were observed at later time points between NK cells growth with (no T cell depl) or without T cells (T cell depl), a delay in the drop of proliferation was observed in NK cells cultured in the presence of T cells (Supplemental Figure 8H), which recapitulates the data observed *in vivo*. These changes in NK cell phenotype correlated with the degree of activation of CD8 T cells during the course of the culture (Supplemental Figures 8I–N), limiting the level of NK cell expansion (Supplemental Figure 8O). Altogether, our findings indicate that anti-PD1 treatment during sustained IL-2 stimulation causes expansion and activation of CD8 T cells that compete with NK cells for the use of stimulating cytokines, therefore delaying the activation and consequently the induction of exhaustion on these innate cells.

## Discussion

Our results suggest that the phenotypical and functional benefits observed after anti-PD-1 treatment on chronically stimulated NK cells are mediated by an indirect effect of anti-PD-1/PD-L1 on CD8 T cells. The activation and expansion of CD8 T cells limit the amount of stimulating cytokines available for NK cells, resulting in a delay of NK cell activation and consequently the induction of exhaustion during prolonged stimulation. These results, therefore, suggest the existence of a delicate balance between CD8 T cells, Tregs, and NK cells, regulated in part by their ability to respond to cytokines. A disruption of this equilibrium influences the homeostatic response to stimuli of each other.

Despite the fact that there was barely any expression of PD-1 in mouse NK cells during IL-2 *in vivo* stimulation, we initially hypothesized that the decrease of the number of NK cells after chronic stimulation could be the consequence of a preferential death of a PD-1-expressing population. PD-1 blockade therapy resulted in a mild improvement of the NK cell activation phenotype after chronic stimulation, but no effect was observed *in vitro*, suggesting that other mechanisms might contribute to the improvement observed after anti-PD-1 *in vivo* administration. This result disagrees with many others that have suggested a role for PD-1 on NCE, a correlation that we could not find in our model (17, 22, 23, 26, 27, 44). Many of these studies were done in human NK cells or infection models that might be more efficient in inducing a PD-1-dependent effect (26, 27, 29, 34). Additionally, Hsu et al. showed an improvement of PD-1/PD-L1 blockade only on tumor-infiltrating NK cells, where higher expression of PD-1 was observed (22). Like our study, NK cells collected from the spleen of the tumor-bearing mouse had low levels of PD-1 (22).

A recently published study has shown in an *in vivo* mouse tumor model that PD-L1 blockade enhances NK cell function and prevents NCE by directly targeting PD-L1^+^ NK cells, identifying a mechanism of NK cell regulation independent of PD-1 (45). We could not identify a direct effect of PD-L1 blockade on NK cells as anti-PD-L1 treatment failed to induce any phenotypical changes on NK cells cultured with IL-2. However, like in this study, we did observe improvement on NK cell activation *in vivo*, which in our case was a process mediated by CD8 T cells. The CD8 T cell compartment was not analyzed in Dong's article and therefore its contribution on the regulation of NCE in the mouse model studied in this paper could not be fully excluded (45).

IL-2 is a pleiotropic cytokine that activates T cells, NK cells, and DC cells. IL-2 binds to IL-2Rs, which display different affinities for the cytokine. CD25 (IL-2Rα) has the highest IL-2 affinity compared to CD32 (IL-2Rγc) and CD122 (IL-2Rβ). CD25 is constitutively expressed on Tregs and thus IL-2 therapy results in an expansion of these cells, which mediates NK and CD8 T cell inhibition through a variety of mechanisms (36, 46–48). Some include the release of immunosuppressive cytokines (IL-10 and TGFβ) upon activation, whereas others require cell-to-cell interactions and act through the activation of checkpoint inhibitors (CTLA-4 and PD-1) causing apoptosis (36). Additionally, Tregs can further contribute to regulate IL-2-mediated immune responses by sequestering IL-2, making it less available to effector cells. Thus, it does not come as a surprise that the depletion of Tregs or the neutralization of IL-10 or TGFβ causes an enhanced expansion of CD8 T cells and NK cells, as previously reported (36, 46, 47). However, the extent to which Tregs depletion affected the activation and expansion of CD8 T cells, while NK cell activation phenotype was somewhat restricted, was interesting. The upregulation of CD25 observed on CD8 T cells upon activation (43) (Supplemental Figure 4E) can indeed provide an advantage over NK cells for IL-2 and explain the differences in the level of activation between both cell types. Similar competition between immune cells has been observed for IL-15 (49).

The existence of regulation between NK and CD8 T cells has been previously reported in many infection models (50–55) and an antigen-independent IL-2 model (36). The results obtained from this study, where a low dose of IL-2 was administered for a short period, indicated a bi-directional regulation between CD8 T and NK cells; yet, NK cells seemed more prone to exert a stronger control on CD8 T cells in a FasL-dependent manner (36). This regulation of NK cells toward CD8 T cells has been previously described as a mechanism to prevent exacerbated CD8 T cell-dependent immune responses and to be critical in the control of immunopathology during mouse cytomegalovirus (MCMV) and lymphocytic choriomeningitis virus (LCMV) infections due to toxic levels of circulating cytokines produced by effector CD8 T cells (52, 53, 56). The control of the infection was implemented by directly eliminating effector cells in an IL-10 and/or perforin-dependent manner (52, 53). A mechanism dependent on NKG2D has also been suggested as a means to eliminate effector CD8 T cells due to upregulation of NKG2D ligands shortly after activation (51, 57, 58). NK cells were also reported to indirectly regulate CD8 T cells by controlling CD4 T cells and DCs (50, 55). Similarly, high levels of PD-L1 on NK cells from tumor-bearing mice limited DC-dependent cross-priming, reducing tumor-specific CD8 T cell priming (59).

CD8 T cells, along with other immune cells such as DCs, can also exert an immune suppressor effect on NK cells due to the upregulation of the NKG2A ligand on CD8 T cells during activation that inhibits NK cells after engaging with NKG2A (54). Notably, the presence of tumor-infiltrated NK cells at the draining lymph nodes of breast cancer patients that express PD-1 and NKG2A has been described (25).

However, targeting NK cells has also shown beneficial effects in the control of persistent infection by facilitating the expansion and activation of T cells. Importantly, to obtain these effects, the timing of NK depletion was crucial (52). Waggoner et al. showed that early NK cell depletion during LCMV infection caused T cell-mediated immunopathological effects, as reported by many others (50–53). In contrast, if NK cell depletion was postponed 2–3 weeks after infection, then an improvement in the control of viral load and in the presence of effector CD8 T cells was seen (52). In this model, the presence of CD4 T cells was important to increase IFNγ^+^ CD8 T cells (52). It was suggested that rescuing dysfunctional CD8 T cells was the mechanism by which a better control of viral load was achieved (52). Additionally, it was also possible that the availability of more stimulating cytokines in the absence of NK cells at that specific time point could promote naïve CD8 T cells to differentiate toward effector cells and contribute to better management of the infection.

In our current and previous studies, we have seen that mouse NK cell dysfunction is observed after ~12 days of prolonged high doses of IL-2, reaching the peak of function and activation shortly after stimulation is started (9). The initial expansion of NK cells after acute IL-2 stimulation is accompanied by a reduction of NK cell numbers during chronic stimulation, a phenomenon that is not observed in Tregs and CD8 T cells. It is important to note that there is a possibility of a conversion of NK cells toward a less functional innate lymphocyte type 1 (ILC1) subset during chronic stimulation as suggested by Gao et al. (35). We believe this is not the case because the analysis is mainly focused on splenic NK cells where the proportion of ILC1 is minimal (1–3% over the total Lin^−^CD49b^+^NKp46^+^ cells) in resting mice when compared to other organs such as the liver or small intestine (60), but we cannot fully exclude this possibility without further analysis.

Nevertheless, in the present study, we show that this NCE phenotype is ameliorated by PD-1/PD-L1 neutralization. Interestingly, the inhibition of PD-1/PD-L1 resulted in enhanced expansion, activation, and function of CD8 T cells. Indeed, CD8 T cells displayed features characteristic of better activation and function such as upregulation of Eomes, T-bet, and NKG2D. Upregulation of a PD1^+^Tim-3^−^ CD8 T cell subset was observed as well during chronic stimulation in anti-PD-1- or anti-PD-L1-treated mice. The fact that CD8 T cells upregulates PD-1 and CD25 upon IL-2 stimulation suggests that PD-1/PD-L1 inhibition might provide an advantage for CD8 T cells over NK cells in the use of IL-2 and other stimulating cytokines.

Some studies have suggested that the Eomes^high^PD-1^high^ CD8 T cell subset, along with the co-expression of other inhibitory checkpoint molecules (BTLA, CD160, and Lag3), represents a subset with a severe state of exhaustion (20, 61). On the contrary, the T-bet^high^PD-1^int^ CD8 T cell population is more responsive and shows enhanced cytokine secretion potential (20, 61). Indeed, T-bet expression, but not Eomes, on tumor-reactive T cells was shown to be expanded after combinatorial checkpoint blockade therapy (62). In accordance with this study, upregulation of T-bet on CD8 T cells was also observed after PD-1 therapy and chronic IL-2 stimulation. However, in our model, this upregulation was accompanied by Eomes expression as well. This subset has shown to be capable of producing higher levels of GranB after anti-PD-1 therapy as described by other studies (20).

The expression of PD-1 on CD8 T cells has also been highly associated with their activation status. Supporting this concept, it has been shown that the adoptive transfer of tumor-infiltrated PD-1^+^ CD8 T cells was capable of containing tumor progression (63). Similarly, an increase of antigen-specific CD8 T cells that express PD-1, but not Tim-3, has been found in the tumor microenvironment after PD-1/CTLA-4 checkpoint blockade therapy (9, 19). In agreement with these studies, an increase of a Tim-3^−^PD-1^+^ CD8 T cell subset was indeed observed after acute and chronic stimulation after neutralization of the PD-1/PD-L1 pathway.

Additionally, similar to NKG2D on NK cells (9, 64, 65), several studies have suggested an important role of NKG2D^+^ CD8^+^ T cells in the battle against tumor and infections (58, 66). Indeed, a recent immunotherapeutic approach that triggers IL-2 signaling in only NKG2D^+^ cells has provided a superior antitumor response (67). Hu et al. showed that NKG2D expression of CD8 T cells was regulated by STAT3 phosphorylation upon CD28 activation (66). This CD28/PD-L1 costimulatory pathway was also shown to be critical for PD-1 therapy success (38), which could be correlated to the enhanced levels of NKG2D on CD8 T cells observed in PD-1-treated mice after chronic stimulation.

Taking these studies into account, our results indicate that PD-1 therapy might provide an activating advantage on CD8 T cells to compete for IL-2, preventing NK cell access of this cytokine. The depletion of CD8 T cells during IL-2 stimulation does, in fact, regress any benefit that PD-1 therapy could have on NK cells after chronic stimulation supporting this hypothesis.

Interestingly, a study has recently shown that IL-2 therapy combined with anti-PD-1 therapy causes an increase of CD8 T cells, but not of NK cells (68). In this study, the authors used an IL-2/anti-IL-2 immune complex (IL-2Cx) with a longer half-life that favors interactions with CD32 and CD122, making IL-2 more readily accessible to CD8 T cells and NK cells (68). When combined with anti-PD-1 therapy in tumor mouse models, the improved anti-tumor efficacy was mediated specifically by increasing the proportion of tumor-infiltrating CD8 T cells, but not tumor-infiltrating NK cells as only the depletion of CD8 T cells resulted in the loss of anti-PD-1/IL2Cx anti-tumoral benefits. Surprisingly, in order to reveal the role of NK cells in the anti-tumor response, Tregs needed to be controlled by anti-CTLA-4 therapy (68).

In spite of the dual regulation described between both CD8 T cells and NK cells, caused by the competition for space and resources, direct or indirect lysis, and functional inhibition, the reality is that both of these populations can also work together to mount a stronger response. Therapeutic approaches involving neutralization of checkpoint inhibitors that requires both CD8 and NK cell responses support this idea of immune-self regulation (21, 22, 69). Moynihan et al. also demonstrated the necessity of both immune populations to eradicate large established tumors treated with combined neutralization of checkpoint inhibitors CTLA-4 and PD-1 (70). Additionally, a recent report has shown the importance of DC and NK cell crosstalk in the CD8 T cell anti-tumor response (71).

Current therapies are searching for the magical weapon that results in a prolonged and stronger anti-tumor response, focused on enhancing the function and presence of effector cells. Preventing immune cell exhaustion has recently become an important target in this search for this perfect immunotherapy. However, a delicate balance between immune cells exists and disruption of this balance might result in unknown effects in the form of toxicities, suppression, competition, or compensatory mechanisms. The results obtained from this study suggest a delay of NCE due to direct competition with CD8 T cells, revealing a positive bystander effect of anti-PD-1 therapy in NK cells despite the absence of a relevant expression of PD-1 in this cell type. This study does also show the importance of evaluating the short- and long-term impact of immunotherapy on any single immune cell component in order to truly understand the implications and exploit the beneficial effects of any immunotherapeutic approach.

## Data Availability Statement

All datasets generated for this study are included in the article/Supplementary Material.

## Ethics Statement

The animal study was reviewed and approved by IACUC at Stanford University and University of Navarra.

## Author Contributions

MA designed and performed research, analyzed data, and wrote the manuscript. FS, JB, AW, and AM contributed in conducting experiments. FS, AP, and PB provided scientific input and assisted with the preparation of the manuscript. RN provided overall scientific guidance and helped write the manuscript.

### Conflict of Interest

The authors declare that the research was conducted in the absence of any commercial or financial relationships that could be construed as a potential conflict of interest.
