# Supplementary material for: Indirect Impact of PD-1/PD-L1 Blockade on a Murine Model of NK Cell Exhaustion
Source: Front Immunol. 2020 Feb 11;11:7. doi: 10.3389/fimmu.2020.00007 (PMC7026672; doi:10.3389/fimmu.2020.00007)

Antibody	Clone	Provider	Catalogue
CD3	145-2C11	BioLegend	100334, 100355
CD11b	M1/70	BioLegend	101259, 101208
CD11c	N418	BioLegend	117334
CD19	6D5	BioLegend	115541
CD25	PC61	BD Bioscience	565314
CD4	RM4-5	BioLegend	100552
CD4	GK1.5	BD Bioscience	612952
CD44	IM7	BioLegend	103006
CD49b	DX5	BioLegend	108906
CD62L	MEL-14	BioLegend	104448
CD8	53-6.7	BioLegend	100714, 100744
CD8	Lyt3.2	BioXcell	BE0223
CD8	53-6.7	BD Bioscience	563795
CD96	3.3	BioLegend	131705
DNAM1	1.00E+06	BioLegend	128803,
Eomes	Dan11mag	BioLegend	12-4875-82
Eomes	Dan11mag	Thermo Fisher Scientific	50-4875-80
Foxp3	MF-14	BioLegend	126419
Granzyme B	GB11	BioLegend	515403
IFNg	XMG1.2	BioLegend	505814, 505838
Ki67	16A8	BioLegend	652420
KLRG1	2F1/KL1261	BioLegend	138409, 138423, 138414
Ly49C/I	5E6	BD Bioscience	557418
Ly49G2	4D11	BD Bioscience	555315, 742885
Ly6G	1A8	BioLegend	127633
Mouse IgG1, Kappa		Thermo Fisher Scientific	50-4714-80
NK1.1	PK136	BioLegend	108720, 108722, 108745, 108748
NKG2A	16A11	Thermo Fisher Scientific	12-5897-82
NKG2A	16A11	BioLegend	142809
NKG2D	CX5	Thermo Fisher Scientific	25-5882-82
NKG2D	CX5	BioLegend	130212
PD-1	RPM1-30	Thermo Fisher Scientific	17-9981-80
PD-1	29F.1A12	BioLegend	135220, 135225
PD-1	RMP1-14	BioXcell	BE0146
PD-L1	10F.9G2	BioXcell	BE0101
PD-L1	10F.9G2	BioLegend	124315
PD-L1	MIH5	Thermo Fisher Scientific	12-5982-82
Rabbit IgG	Polyclonal	Abcam	Ab171870
Rabbit IgG XP®	DA1E	Cell signaling Technology	3900S
Rat gamma globulin		Jackson ImmunoResearch	012-000-002
Rat IgG1, Kappa	RTK2071	BioLegend	400418, 400443
Streptavidin		Thermo Fisher Scientific	46-4317-82

Streptavidin		BioLegend	405229
T-bet	4B10	BioLegend	25-5825-80
TCRb	H57-597	BioLegend	109243, 109226, 109241
Thy1.2	30-H12	BioLegend	105324, 105331, 105343
TIGIT	1G9	BioLegend	142105
Tim3	RMT3-23	BioLegend	119706, 119715

Supplemental Table 1. Antibodies for flow cytometry. The table lists all antibodies used in this study, including clone (when applicable), provider and catalogue number.

SUPPLEMENTAL FIGURE LEGENDS

Supplemental Figure 1. Distribution of PD-1 and PD-1 Ligands after chronic stimulation with IL-15 and Poly I:C. C57BL/6 mice were chronically stimulated with IL-15 or poly I:C as previously described (Alvarez *et al.*, 2019, *JCI Insights*). **(A-B)** Representative dot plots for PD-1 and PD-L1 expression are shown on gated NK cells ($CD19^{-}TCR\beta^{-}NK1.1^{+}$) after IL-15 or poly I:C stimulation. Data is representative of three independent experiments with 3-4 mice per group.

Supplemental Figure 2. Chronic IL-2 stimulation alters NK cell proliferation and function. **(A)** Representative histograms and total percentage of Ki67 after IL-2 treatment are shown for gated NK cells. **(B)** Representative histograms and the total percentage of GranB are shown for NK cells ($CD3^{-}CD49b^{+}$) that were further stimulated for 4h with anti-NK1.1. **(C)** Representative dot plots and the total percentage of IFNγ production of IL-2 treated NK cells ($CD3^{-}CD49b^{+}$) after NK1.1 stimulation. Data are representative of five independent experiments with 3-4 mice per group (mean ± SEM). One-way ANOVA was used to assess significance. Significant differences are displayed for comparisons with the acute group (**p<0.01, ***p<0.001).

Supplemental Figure 3. PD-1 blockade augments the activating phenotype of NK cells without affecting the expression of the inhibitory receptors after chronic IL-2 stimulation. (A) Percentage of the variance for each or cumulative principal component (PC) is shown from the PCA analysis (Fig.2A). **(B)** Representation of the NK cell markers that drive PC1 and PC2 are shown. **(C)** Representative dot plots of the activating marker Ly49G2 and Thy1.2 are shown for gated NK cells. **(D-F)** The total percentage of NK cell activation markers (Ly49G2, Thy1.2, and DNAM1) is shown on gated NK cells. **(G-I)** The percentage of NK cell inhibitory receptors (NKG2A, TIGIT, and CD96) is shown on gated NK cells. **(J)** The percentage of lysis is shown for a CFSE-based killing assay against the NK cell sensitive cell line Yac-1 of treated splenocytes cultured for 4h at different E:T ratios. **(K)** The level of IFNγ of the serum of treated mice is shown. Data is representative of three independent experiments with 3-4 mice per group (mean ± SEM). Two-way ANOVA was used to assess significance. Significant differences are displayed for comparisons with the rIgG-treated group (*p<0.05, **p<0.01, ***p<0.001).

Supplemental Figure 4. Blockade of PD-L1 does not directly influence the progression of NK cells exhuasiton in vitro. NK cell were cultured as in figure 3 in the presence of anti-PD-L1 or isotype control. **(A-C)** Representative histograms, the total percentage or MFI of PD-L1 is shonw for gated NK cells or PD-L1$^+$ NK cells (C). **(D-F)** MFI expression of Eomes, T-bet, and NKG2D is shown for Eomes$^+$, T-bet$^+$ or NKG2D$^+$ NK cells respectively. **(J-K)** The percentage of KLRG1 or Ki67 is shonw for gated NK cells. Data is representative of two independent experiments done by triplicate (mean ± SEM). Two-way ANOVA was used to assess significance. There is no significant differences.

Supplemental Figure 5. NK cells stimulated in the absence of Tregs show sings of early exhaustion after acute IL-2 treatment. Analysis of NK cells was done as in DTR-Foxp3 transgenic mice as in figure 5. **(A-B)** The total percentage of NKG2D (A) and KLRG1 (B) is shown on gated NK cells. Data is representative of two independent experiments with three mice per group (mean ± SEM). Two-way ANOVA was used to assess significance (**p<0.01, ***p<0.001).

Supplemental Figure 6. Blockade of the PD-1/PD-L1 pathway does not alter the distribution of effector CD8 T cells. (A) Phenotypic distribution of naïve ($CD62L^{+}CD44^{-}$), central memory ($CD62L^{+}CD44^{+}$) and effector memory ($CD62L^{-}CD44^{+}$) CD8 T cells is shown. **(B)** Representative dot plots of NKG2D and CD25 expression on gated $CD44^{+}$ CD8 T cells to show the changes on the bystander non-antigen specific $NKG2D^{+}CD25^{-}$ CD8 T cell subset and the expression of CD25 after anti-PD-1 treatment during IL-2 stimulation. Data is representative of three independent experiments with 3-4 mice per group.

Supplemental Figure 7. CD8 T cell depletion efficacy during chronic IL-2 stimulation. Representative dot plots of the expression CD8 and CD4 on gated $CD19^{-}TCR\beta^{+}$ T cells are shown. The anti-CD8β mAb (clone Lyt3.2) was used for depletion, and the anti-CD8α mAb (clone 53-6.7) was used for detection by flow cytometry.

Supplemental Figure 8. NK cell activation by in vitro long-term IL-2 stimulation is impair by the presence of mature T cells in the culture. Thy1.2- (T cell deple) or untouched (No T cell depl) BM/spleen derived cells were cultured as described in figure 3. Adherent and non-adhirent cells were collected and analyzed for NK cell and CD8 T cell phenotype by flow cytometry. **(A-B)** Representative dot-plots and total percentage of IFNγ producing NK cells. **(C)** Total percentage of NK cells making GranB. **(D-H)** Changes over NK cell exhaustion parameters (Eomes, T-bet, NKG2D, KLRG1 and Ki67) are shown. **(I-J)** Representative histograms and total percentage of GranB producing cells is shown for gated CD8 T cells in the no T cell depleted group. **(K-M)** Total percentage of Eomes, T-bet and Ki67 is shown on gated CD8 T cells in the no T cell depleted group. **(N)** The total percentage of CD8 T cells is shown at different time points of IL-2 in vitro cuture of Thy1.2+/- cells. **(O)** The total percentage of NK cells at different time points is shown in T cell depleted and no T cell depleted cultures. Data is representative of two independent experiments done by triplicate (mean ± SEM). Two-way ANOVA was used to assess significance. Significant differences are displayed for comparisons with the rIgG-treated group (*p<0.05, **p<0.01, ***p<0.001).

Supplemental Figure 1

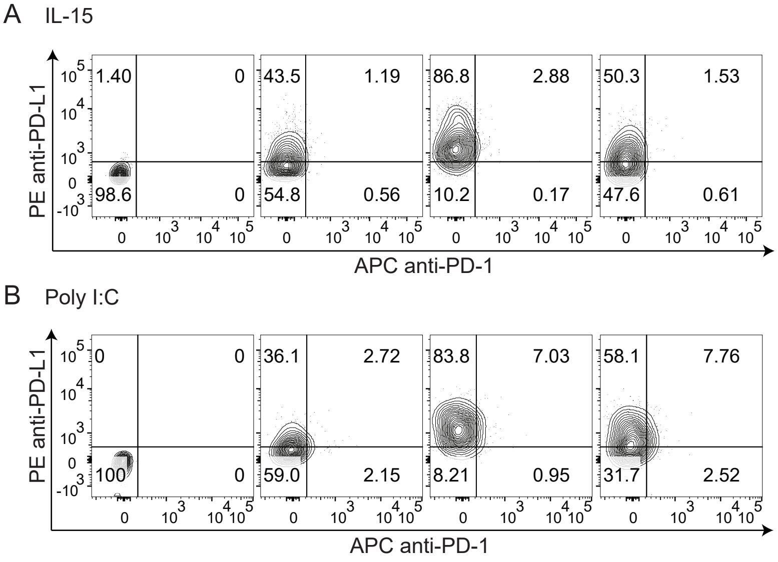

Supplementary Figure 2

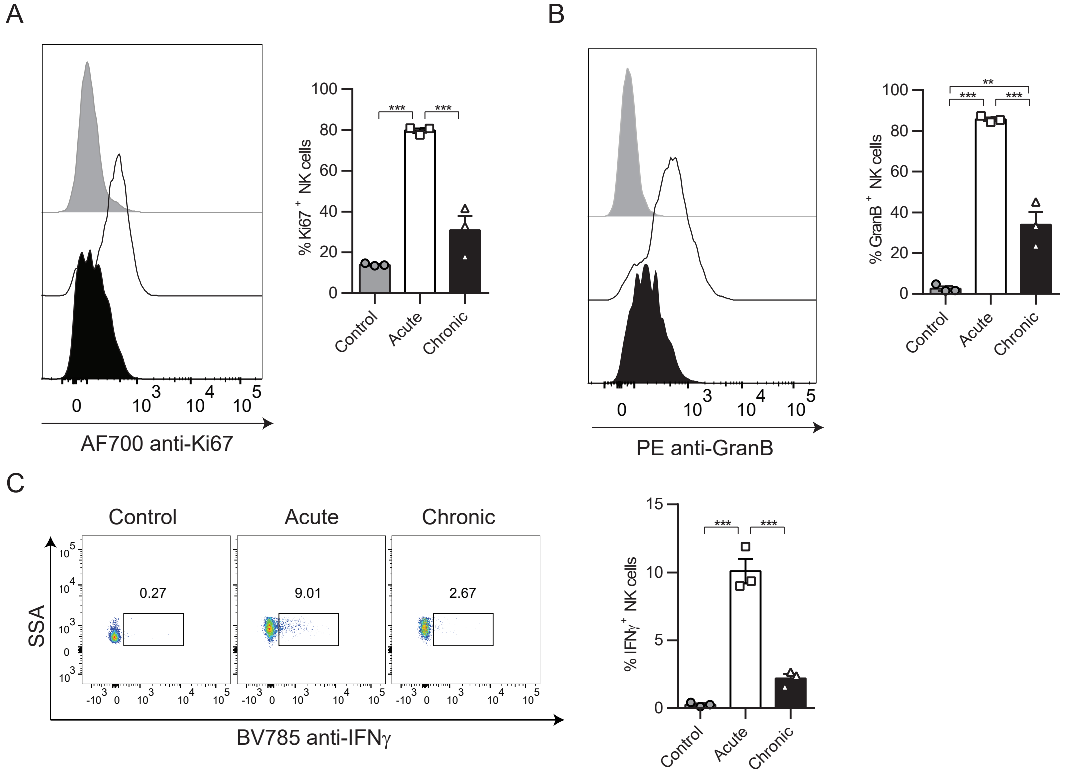

Supplemental Figure 3

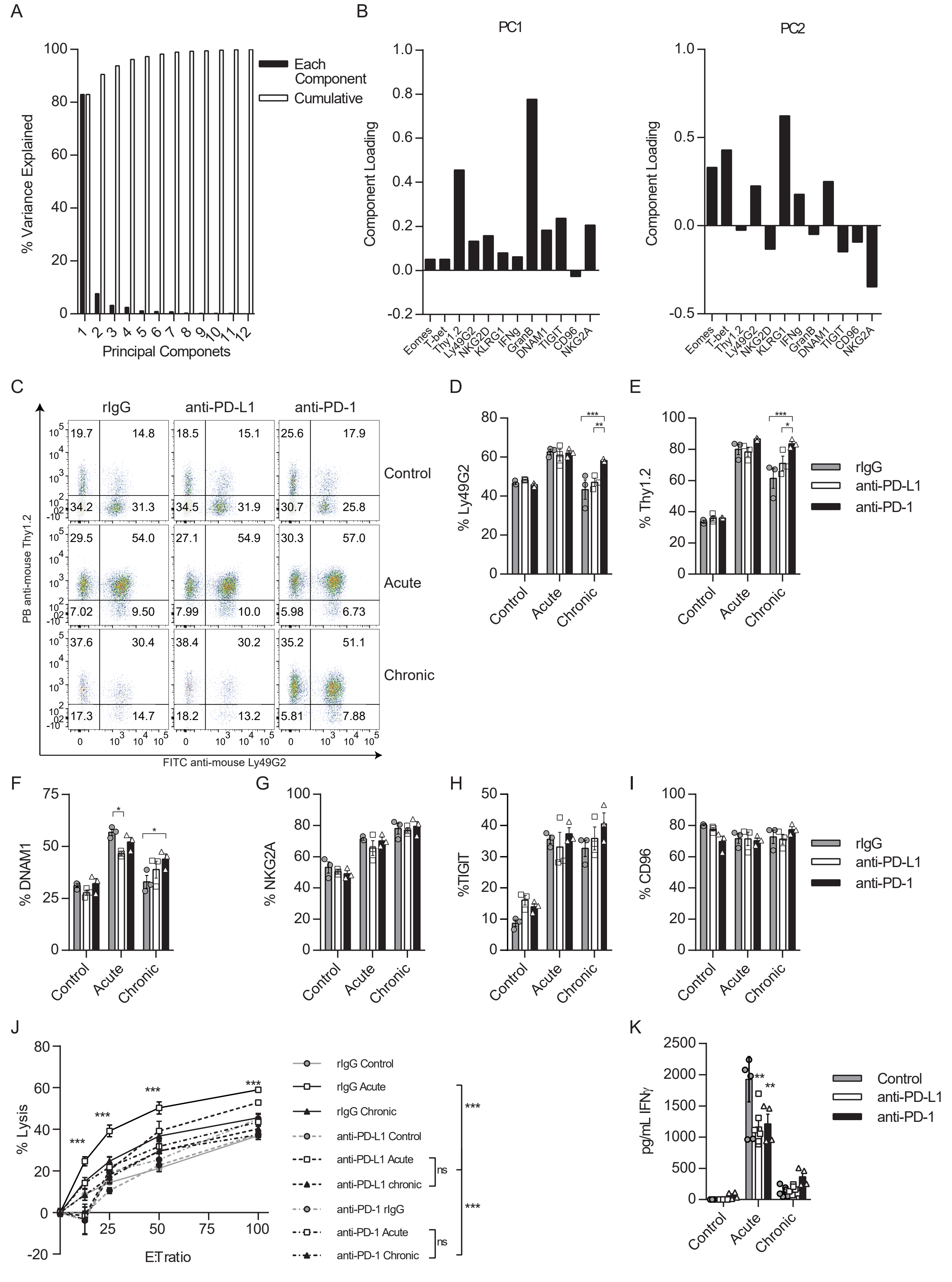

Supplemental Figures 4

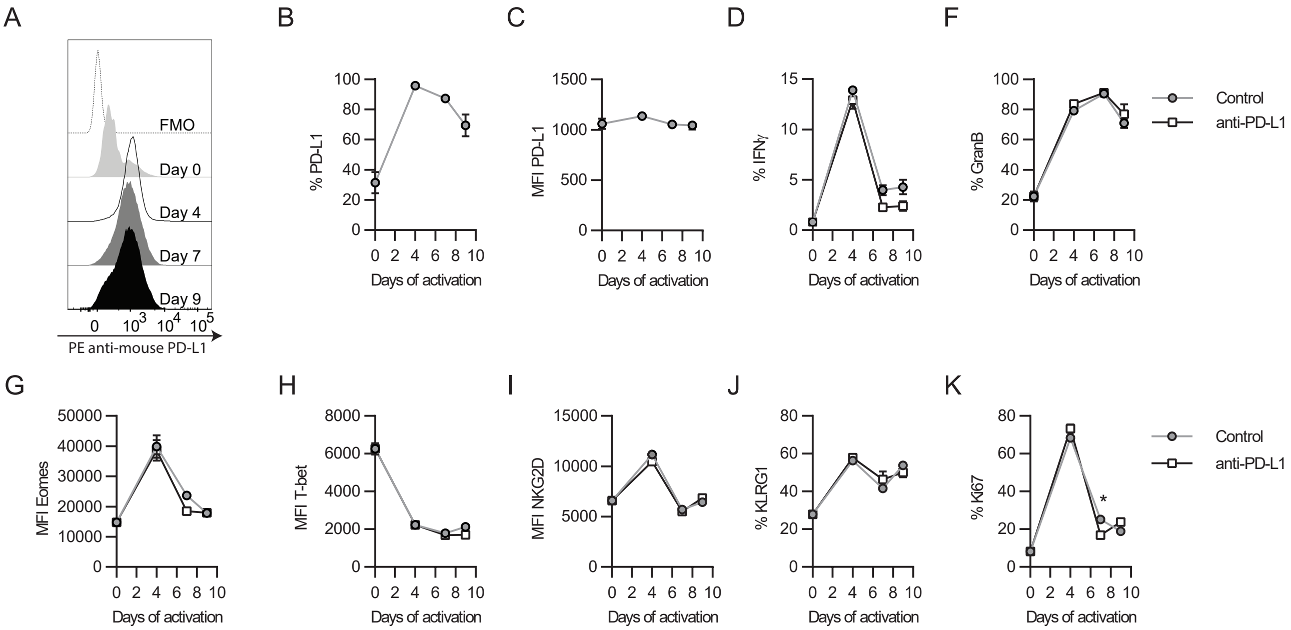

Supplemental Figure 5

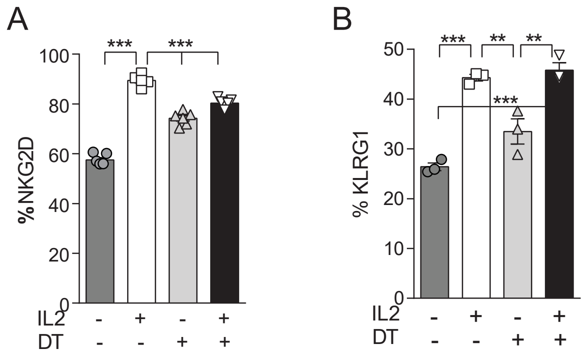

Supplementary Figure 6

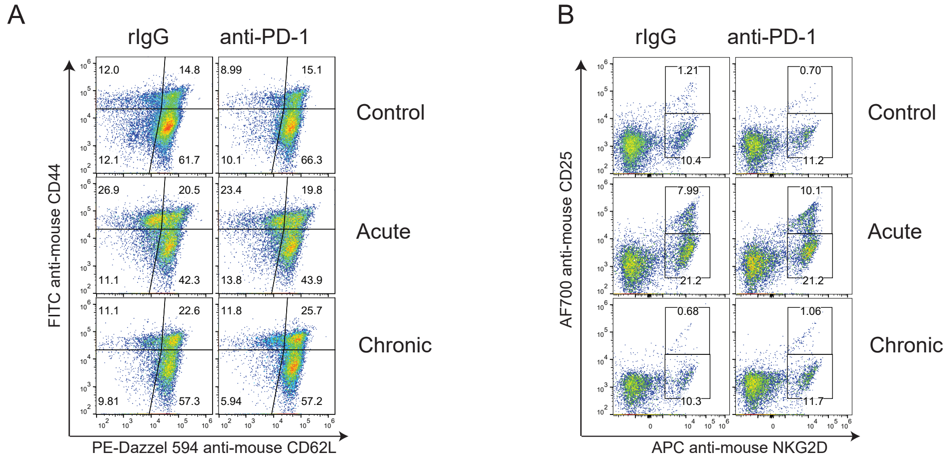

Supplementary Figure 7

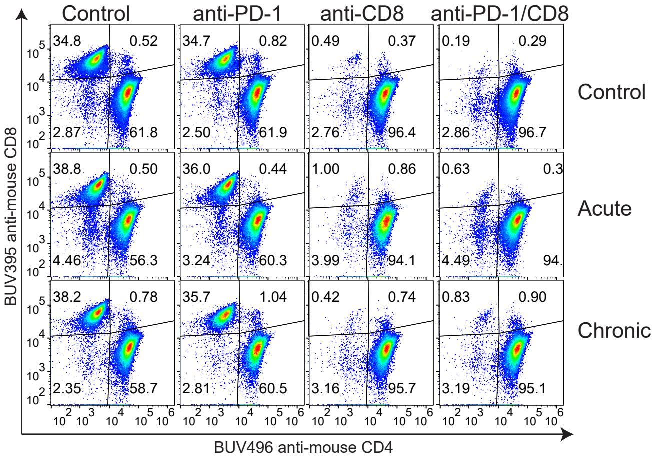

Supplemental Figure 8

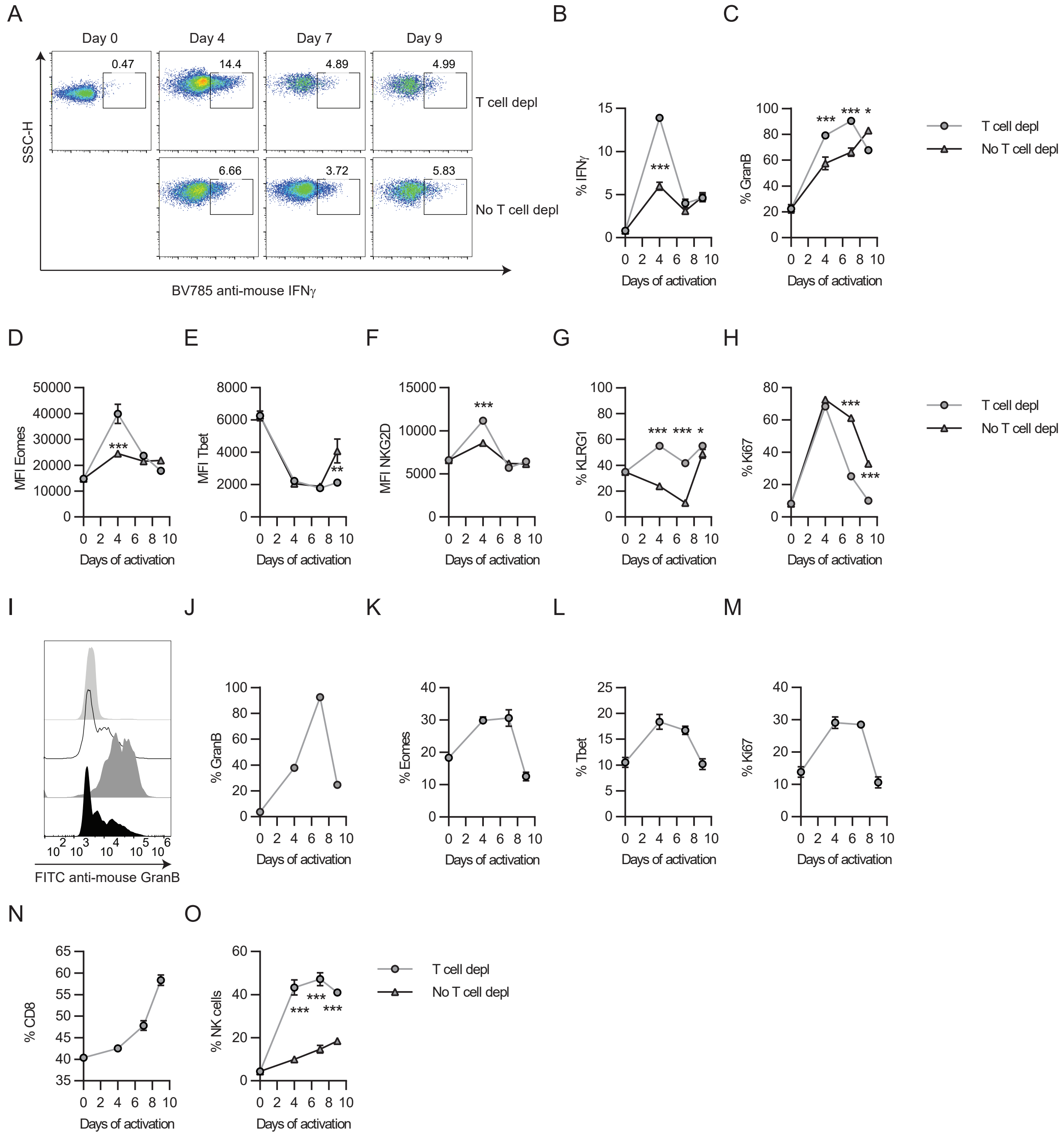

Supplement: Supplementary file 1 [file Data_Sheet_1.pdf]
